# Invasion complexity at large spatial scales is an emergent property of interactions among landscape characteristics and invader traits

**DOI:** 10.1371/journal.pone.0195892

**Published:** 2018-05-17

**Authors:** Ranjan Muthukrishnan, Adam S. Davis, Nicholas R. Jordan, James D. Forester

**Affiliations:** 1 Department of Fisheries, Wildlife, and Conservation Biology, University of Minnesota, St. Paul, Minnesota, United States of America; 2 Global Change and Photosynthesis Research Unit, USDA-ARS, Urbana, Illinois, United States of America; 3 Department of Agronomy and Plant Genetics, University of Minnesota, St. Paul, Minnesota, United States of America; University of Waikato, NEW ZEALAND

## Abstract

Invasion potential should be part of the evaluation of candidate species for any species introduction. However, estimating invasion risks remains a challenging problem, particularly in complex landscapes. Certain plant traits are generally considered to increase invasive potential and there is an understanding that landscapes influence invasions dynamics, but little research has been done to explore how those drivers of invasions interact. We evaluate the relative roles of, and potential interactions between, plant invasiveness traits and landscape characteristics on invasions with a case study using a model parameterized for the potentially invasive biomass crop, *Miscanthus* × *giganteus*. Using that model we simulate invasions on 1000 real landscapes to evaluate how landscape characteristics, including both composition and spatial structure, affect invasion outcomes. We conducted replicate simulations with differing strengths of plant invasiveness traits (dispersal ability, establishment ability, population growth rate, and the ability to utilize dispersal corridors) to evaluate how the importance of landscape characteristics for predicting invasion patterns changes depending on the invader details. Analysis of simulations showed that the presence of highly suitable habitat (e.g., grasslands) is generally the strongest determinant of invasion dynamics but that there are also more subtle interactions between landscapes and invader traits. These effects can also vary between different aspects of invasion dynamics (short vs. long time scales and population size vs. spatial extent). These results illustrate that invasions are complex emergent processes with multiple drivers and effective management needs to reflect the ecology of the species of interest and the particular goals or risks for which efforts need to be optimized.

## Introduction

The spread of invasive species is a global challenge that threatens both losses of natural biodiversity [1] and significant economic costs [2,3]. As such, invasive spread should be considered when novel biomass crops are being introduced to diversify agroecosystems and produce biomass feedstocks for biofuels and other bioproducts. Targeted introductions of these crops in agroecosystems can enhance agricultural production, enhance renewable energy production, and improve conservation of soil, water and biodiversity [4]. However, these introductions may lead to invasions into new habitats, as many traits of productive biomass crops are congruent with those of highly invasive species [5]. Understanding the risks of translocation or introduction of species for agricultural, commercial, biocontrol, or other reasons is a critical need, but remains a difficult challenge. While invasion dynamics can be described in simple, idealized models [6–8] understanding invasions in realistic systems with more complex landscapes and species interactions is a far more challenging goal [9,10]. In order for analyses of the potential risks related to novel crop introductions to be meaningful, they need to account for the role of realistic landscapes and be flexible enough to reflect the particular characteristics of the species. However, such an effort could additionally provide insights into basic invasion biology [11] while also informing, at realistic scales, practical management questions for the production of novel crop species.

Given their potential for invasion, incorporation of biomass crops into agroecosystems may require a careful balancing of escape and invasion risks with the potential benefits of those crops. Unlike other invasive species, management of these crops will not be focused on eradication or reduction of abundances, but rather maintenance of populations within the constrained spatial boundaries of their agroecosystems. This particular scenario, where a constant source of the invader population will persist, presents a situation where invasion dynamics need to be explicitly considered in the context of broader landscapes. The speed, extent, and success of invasions are in large part influenced by the particular physiological and ecological traits of the invading species (e.g., high fecundity, low resource requirements, broad environmental tolerances, and high dispersal ability [5,7,12]). At the same time, the landscapes in which invaders are introduced also influence invasion dynamics [10]. Fundamentally, different habitats provide better or worse conditions for the invaders, while the extant communities in those habitats may be more or less resistant to invasion. Thus, certain habitat types may be more resistant to invasions, particularly when the abundance of suitable habitat or the growth rates of invader populations are low enough for stochastic local extinctions to have a strong influence on overall invasion patterns.

Moving beyond individual locations to larger spatial scales, the arrangement of different habitat types, each with different suitability for, or biotic resistance to, invaders, can have even more complex impacts on invasion dynamics. While less well established than the importance of invader traits, previous work has identified that landscape characteristics can influence invasive spread, in particular fragmentation [13,14], heterogeneity [12,15], and the presence of dispersal corridors [12,16]. Consideration of landscape metrics indicative of these patterns, such as average patch number, patch diversity, edge density and perimeter to area ratios (for fragmentation and heterogeneity), and the presence of roads (as dispersal corridors) could provide strong indicators of invasion risk. However, even when the influence of landscape characteristics on invasions is considered, it is generally in the context of a single species (e.g., [17,18]). Because each invasive species has a different suite of ecological traits, it is not clear whether landscape characteristics have general effects on invasion risk. As a result, there is a lack of certainty as to how specific landscape characteristics will influence invasions, especially across invaders with different ecologies [12].

In particular, the ability of landscape characteristics to facilitate or restrain invasions also depends on the specifics of the invader [14,19]. While certain organismal traits may be generally considered more or less “invasive” [5], their impact may be contingent on, and should be evaluated in, the context of specific combinations of landscapes and species traits [19,20]. For example, low resource requirements and a fat-tailed dispersal kernel may provide minimal invasive benefit in areas of contiguous, resource-rich habitat; however, these same traits may allow invasion in fragmented landscapes with large areas of low-resource matrix between more productive patches [12,21]. What environmental conditions provide good habitat for an invasive species, and what resource patterns are considered fragmented are all relative to the spatial and temporal scales at which a given species perceives and interacts with those landscape features [22]. Indeed, we contend that a better understanding of these aspects of this “landscape ecology of invasions”, i.e., how the ecological traits of invaders interact with the spatial structure of resources, is critical for predicting and managing the dynamics of an invasion [19,23].

Understanding the role of landscape factors in invasion dynamics is a challenge in part because of the difficulty of identifying and evaluating the structure of real landscapes in a rigorous and comparable way. While numerous metrics have been developed to describe landscape structure [24], these statistics themselves present additional challenges for subsequent analysis and interpretation [25]. Analyses based on landscape metrics require dealing with large numbers of highly correlated variables for which *a priori* expectations of ecological importance may not be obvious [26]. Further, experimentation at the landscape scale is challenging because of the difficulty of sampling or manipulating large areas, and because “equivalent” landscape sections may be generally difficult to find [27]. In these cases, landscape-scale simulation models provide an ideal opportunity to develop and test initial hypotheses about how different metrics of landscape structure interact with species plant traits to affect invasion dynamics.

Here we use such a model, initially parameterized for the potentially invasive biomass crop *Miscanthus* × *giganteus* [18,28], as a case study to explore how hypothetical invasions could unfold in real landscapes from across the United States. Using this model we simulated invasions in a broad range of landscapes to evaluate how invasion dynamics change between landscapes and the influence of particular landscape characteristics on invasions. Additionally, we conducted replicate simulations on each landscape while modifying specific traits of the invader (dispersal ability, ability to use dispersal corridors, establishment ability, and population growth rate) to explore how relationships between landscape characteristics and invasion dynamics change with invader traits. Based on previous work (e.g., [19]) we expect that invader traits likely have stronger effects on invasion dynamics than landscape characteristics, but that there will be high variability and context dependence such that there are certain landscapes where invader traits will not be key drivers of invasion patterns. We note that the particular estimates from our study may not be applicable to all invasive plants in general, but rather they are focused on an herbaceous perennial biomass crop, a plant life history that is of much interest for diversification of agroecosystems and production of plant biomass [4]. Despite the model’s focus on such crops, our study uses *M*. × *giganteus* as an exemplar to address the broader question: can interactions between landscape characteristics and invader traits have strong effects on invasion dynamics?

## Methods

To evaluate the role of landscape characteristics on the dynamics of invasive spread, we simulated invasions across a range of landscapes using an existing model of the potentially invasive biomass species *Miscanthus* × *giganteus* [18]. We repeated simulations on each landscape while shifting parameter values in the model to reflect invaders similar to *M*. × *giganteus* but with different strengths of specific traits related to invasiveness. The results of these simulations were analyzed in a two-step process that first identified how landscape metrics affected invasion rates for each set of invader traits and then explored how the strength of those effects varied as the levels of those invader traits changed.

### Simulations

The *Miscanthus* simulation model we used [18] allowed a founding population of an invader to begin in the center of the landscape and then grow through local reproduction and dispersal. Our simulations were similar to those in the original model in that the habitat type at each position in the landscape, which acts as a proxy for both abiotic habitat quality and the resistance to invasion by the extant community, affected the population growth rate and carrying capacity of the invader; however, we also allowed for the habitat type to influence the probability of dispersing seeds establishing in an empty patch. There was no reduction in the best habitat (grasslands) and the probability of establishment was reduced in other habitats proportionally to the relative values of their habitat expansion rates (*r*_*i*_; the rate of population growth for an individual patch of a given habitat type).

We ran simulations on 1000 landscapes that were randomly selected from the METALAND database [29]. METALAND utilizes data from the US National Land Cover Database (NLCD; [30]) and splits the entire country into a grid of 6.48km x 6.48km landscape tiles. Each tile provides a landscape of 216 x 216 cells, with each cell being 30m x 30m. The only constraints on landscape selection were that the entire landscape was classified (i.e., landscape tiles at the edges of the NLCD coverage area that had empty pixels were excluded) and more than half of the landscape was terrestrial (i.e., not open water). While we used the same model structure as in our previous work, we allowed specific parameters to vary in order to represent hypothetical invaders with similar properties (such as fecundity and relative preference/suitability for different habitat types), but that encompassed a range of levels of certain traits associated with invasiveness. In particular, we varied values of 4 traits: dispersal ability, establishment ability, population growth rate, and ability to use dispersal corridors. To vary dispersal ability and population growth rate, we modified the mean values of the log-normal dispersal kernel and the habitat-specific expansion rates to range from one-third to double the values included in the original model (0.33, 0.5, 1, 1.5, 2). To vary establishment ability, we allowed the frequency with which accessible patches arose to be 0.005, 0.01, 0.025, or 0.05 (the original model used a value of 0.03). Finally, we modified corridor usage by changing the proportion of seeds that actually move along a corridor (given that they landed in corridor cells, here defined to be roads) from 0 to 100% (0, 25, 50, 75, 100%); this value was 50% in the original model.

Simulations for each unique parameter combination (500 total combinations) were run on all of the 1000 landscapes, with initial populations of 50 2^nd^ year plants in each of the 9 cells at the center of the landscape. Simulations were run with annual time steps to simulate 100 years of invasion dynamics. This provided 500,000 total simulations, each with 100 time steps. To capture different aspects of invasion dynamics, four separate response metrics were calculated. To evaluate short term, transient dynamics that reflect earlier stages of invasion, we recorded the yearly population growth rate averaged across the landscape and calculated the mean growth rate over the first 30 years of the simulation (growth rate). Additionally, to evaluate the rate of spread, we recorded the yearly increase in the proportion of landscape cells that had at least one adult plant and the mean across the first 30 years was calculated (expansion rate). Finally, to consider longer-term dynamics, we recorded the final population size averaged across the landscape at the end of the simulations (final population) and the proportion of the landscape with at least one adult plant (final extent); however, even at the end of 100 years, simulations did not reach equilibrium conditions.

### Analysis of landscape characteristics

For each landscape we collected the habitat data from the original METALAND landscape but reclassified the map to account for habitat types that we combined [18] and calculated the proportion of the landscape in each of 11 categories (grassland, pasture, crop, deciduous forest, mixed forest, coniferous forest, wetland (riparian), shrubland, road, water, and unsuitable). Using these reclassified landscapes we calculated landscape-level spatial statistics for area, edge, shape, aggregation, and diversity metrics using FRAGSTATS and details of specific metrics can be found in the related documentation [24]. However, we did not calculate core area or contrast metrics because we had no strong *a priori* standards for edge depth or relative strength of contrast between habitat types. These metrics offer a detailed but generalizable description of many aspects of landscape structure including fragmentation, habitat heterogeneity and diversity, patch shape, and landscape connectivity [24,31].

To reduce the number of modeled landscape metrics in our analysis, we removed all metrics that showed little to no variation across the dataset; these were essentially trivial characteristics (e.g., total area, which was constant across all landscapes). Additionally, for characteristics that were based on a distribution of values from across the landscape (e.g., patch area) we limited the number of parameters used to describe the distribution to three. We only included median, area-weighted mean, and standard deviation metrics to capture information about the central tendency and variability of all patches, as well as characteristics of the most influential patches, while limiting parameters likely to be highly correlated (e.g. median and mean, or standard deviation and coefficient of variation). This led to a total of 50 landscape metrics, 11 of which reflected the composition of the landscape (the abundance of different habitat types) and 39 that described landscape-level spatial structure and patterning; these are hereafter referred to as composition and spatial structure metrics, respectively.

### Statistical analyses

We conducted two levels of statistical analyses: 1) using a Bayesian lasso regression [32] we estimated the influence of each landscape characteristic on invasion for each unique combination of invader traits and response metric, and 2) using the parameter estimates from the lasso regressions, we estimated how the relative importance of landscape characteristics change with respect to the strength of specific invasive traits. The use of lasso regression provides an efficient method to deal with many highly correlated variables for which there are no clear expectations as to relative importance by allowing a shrinkage parameter to reduce the influence of uninformative variables [33].

We used a fully Bayesian approach to generate Markov Chain Monte Carlo (MCMC) samples of the posterior distributions of regression parameters. To fit the models, we used data from 900 of the simulations, and held the remaining 100 out for validation. Using a standard, generalized linear model framework with vague priors, the posterior of the regression model was:

$$P(\beta_{0},\beta ,\sigma |y,X,\tau )\propto \prod_{i=1}^{900}normal({y_{i}|\tau ,X_{i};\beta }_{0},\beta ,\sigma )\times \prod_{j=1}^{50}Laplace(\beta_{j}|0,\tau )\times normal(\beta_{0}|0,0.01)\times uniform(\tau |0.001,30)$$ (Eq 1)

where ***y*** is the values of the particular invasion metric of interest and *β*_*1*_ to *β*_*50*_ indicate coefficients for each of the 50 landscape characteristics which are quantified for each unique landscape in the matrix **X**. For each data set and response variable, we centered and scaled all independent variables and then ran three MCMC chains for 10,000 iterations after a burn-in of 5,000 iterations. We checked chains for convergence by visually checking the trace plots and calculating Gelman and Rubin’s convergence statistic. While sampling the posteriors for each model, we recorded the ability of the model to predict out-of-sample data (i.e., the results of simulations on the 10% of landscapes not included in the regression). This process was repeated for 24 different values of the rate parameter of the regression coefficients’ Laplace prior (τ ranging from 0 to 2,000); this rate parameter controls the degree to which parameters are shrunk towards 0. By plotting the predictive ability of models fit at each level of τ, we were able to determine the optimal degree of parameter regularization without imposing arbitrary model selection on our independent variables. All analyses were conducted with JAGS (Just Another Gibbs Sampler) using rjags [34] in the program R, version 3.1.2 [35].

After estimating β coefficients for each landscape characteristic at each unique combination of invader traits with the lasso regression we then evaluated how the importance of those landscape characteristics for predicting invasion dynamics changed with invader traits. We used the posterior means of parameters from the optimum lasso regressions as response variables in a standard Bayesian generalized linear model with the values of each of the invader traits as the independent variables in the regression. Here, our posterior was simply:

$$P(\Phi ,\lambda |\hat{\beta },Z)\propto \prod_{i=1}^{500}normal(Z_{i},|\hat{\beta_{i}};\Phi ,\lambda )\times \prod_{j=1}^{4}normal(\Phi_{j}|0,0.001)\times normal(\lambda |0,0.001)$$ (Eq 2)

where $\hat{\beta }$ is the estimated coefficients of given landscape characteristic from above, *λ* is the intercept for the linear predictor, and ***Φ*** is the coefficient for each of the invasiveness traits and **Z** is a matrix of the values of each of the invasiveness traits in each set of simulations. As with the lasso regression, we ran three MCMC chains for 10,000 iterations after a burn-in of 5,000 iterations and checked for convergence using standard methods. We calculated the mean and 95% credible intervals of the posterior distribution of each regression coefficient and considered Φ whose credible intervals did not include 0 to indicate a meaningful relationship between the plant invasiveness trait and the importance of the landscape characteristic on invasions.

## Results

**Fig 1:**
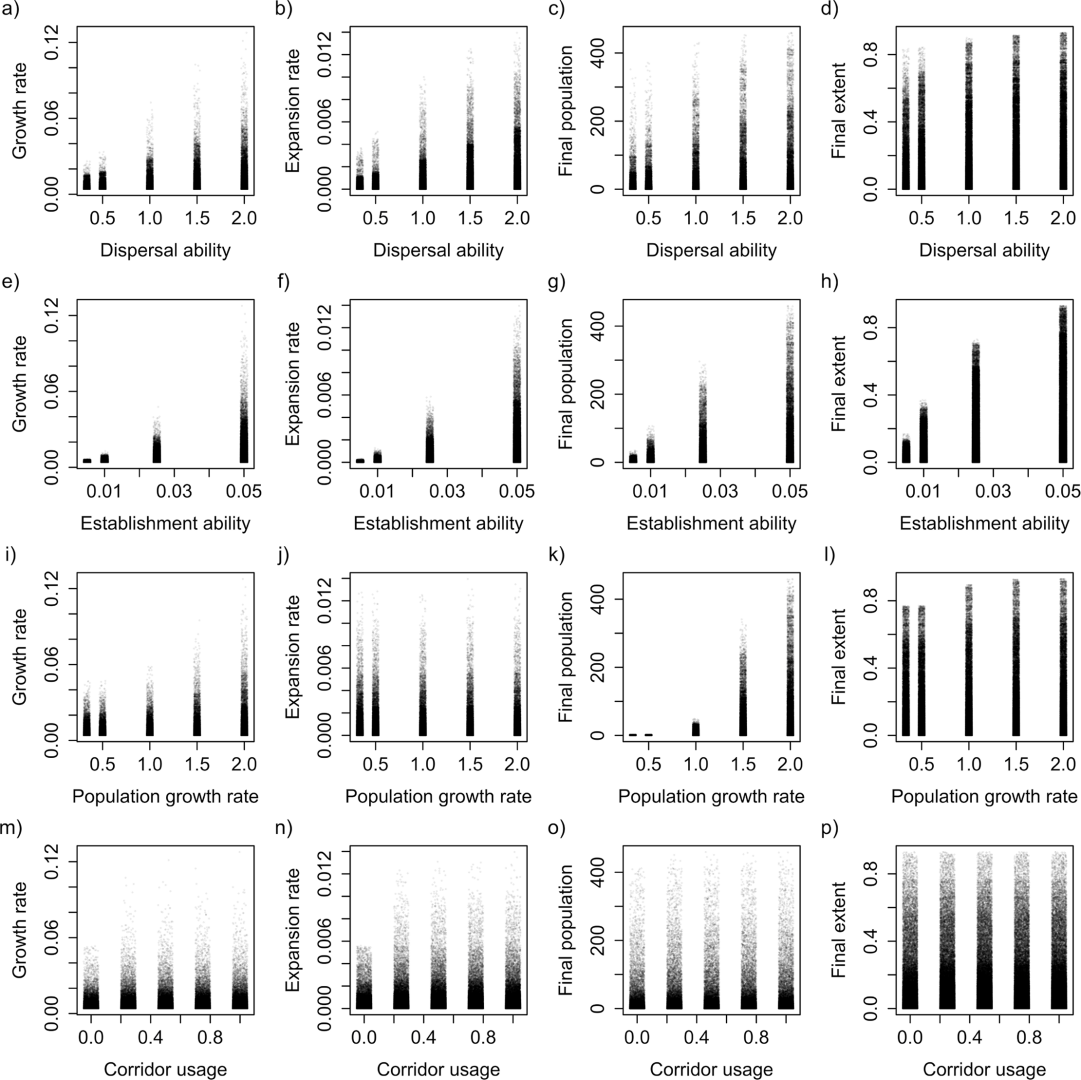
Individual panels show the simulation results for each of the 4 response metrics (expansion rate in the first panel of each row, growth rate in the second, final extent in the third and final population in the fourth) across different values of each of the 4 invasiveness traits (dispersal ability in the top row, corridor usage in the second, establishment ability in the third, and population growth rate in the fourth).

Outcomes of invasion simulations displayed a wide variety of outcomes, ranging from essentially no invasive spread to extensive infestations with millions of individuals across the landscape and spread rates of nearly 1% of the entire landscape being newly colonized every year. In general, across all invasion metrics (30 year average expansion rate, 30 year average population growth rate, final invasion extent and final population size), increasing strength of invasiveness traits had a strong, positive relationship with invasion magnitude (validating them as traits that support invasiveness), which also corresponded with an increase in variability (Fig 1). However, the dispersal corridor usage trait was an exception as increasing its level from zero yielded an initial increase in invasion magnitude, while at the highest level there was a slight reduction, suggesting a nonlinear relationship with a maximum at an intermediate usage level.

**Fig 2:**
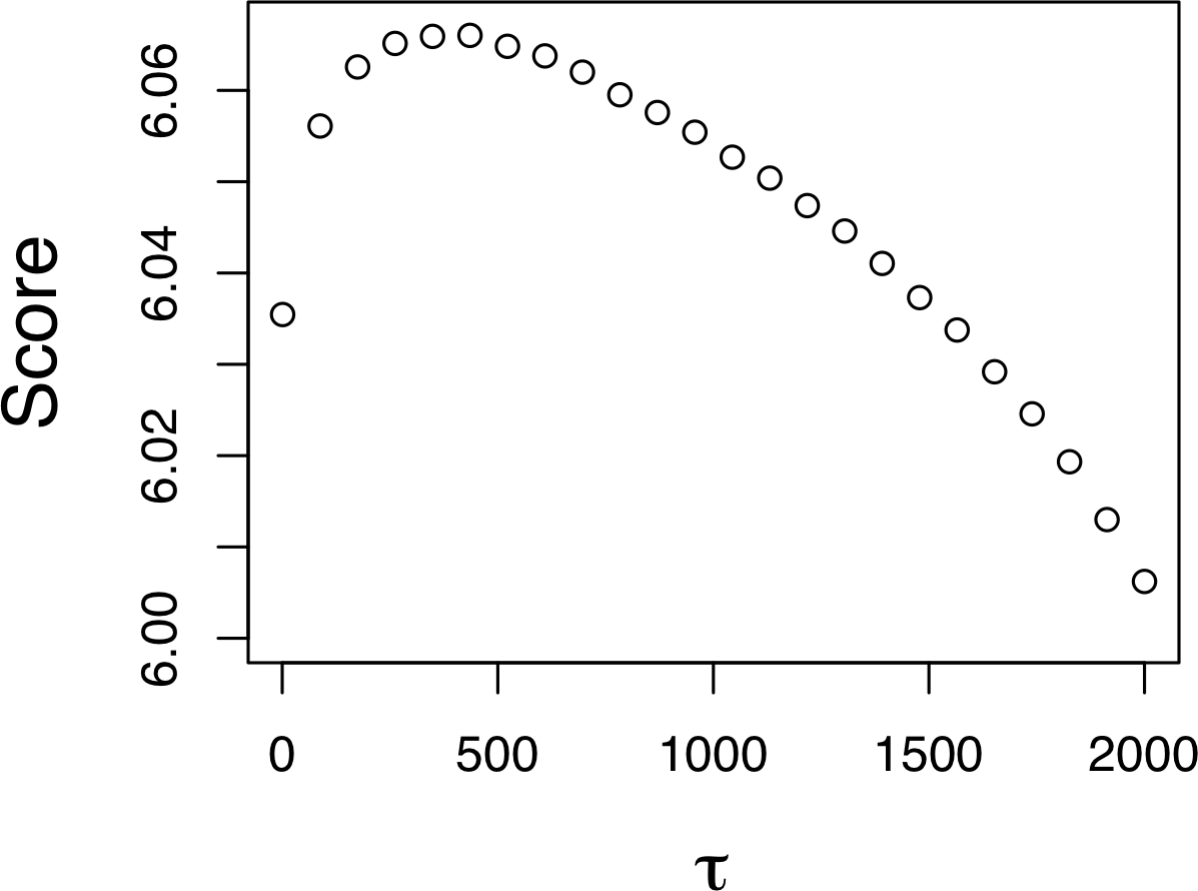
Results of an example lasso analysis for one set of invasiveness parameters indicating the optimal shrinkage value (τ in the Laplace distribution) for parameter regularizations. The score value is the log predictive density of the model for the out of sample data. The data shown are only for the regularization for the parameters that most closely align with the empirical invasiveness traits for *M*. × *giganteus*.

Lasso regressions for each of our unique combinations of invader traits selected an intermediate λ value (indicating that we identified an actual optimal value for the lasso width, as seen in Fig 2) in 78% of the scenarios (79%, 76%, 92%, and 64% for transient expansion, transient growth, final extent and final population respectively). Conditions where the smallest λ value provided the best fit were asymptotic and thus a larger range of values would not have led to substantially better model fits. Those conditions represented scenarios with very low invasion rates across all landscapes, and thus the best models were driven almost entirely by β_0_.

**Fig 3:**
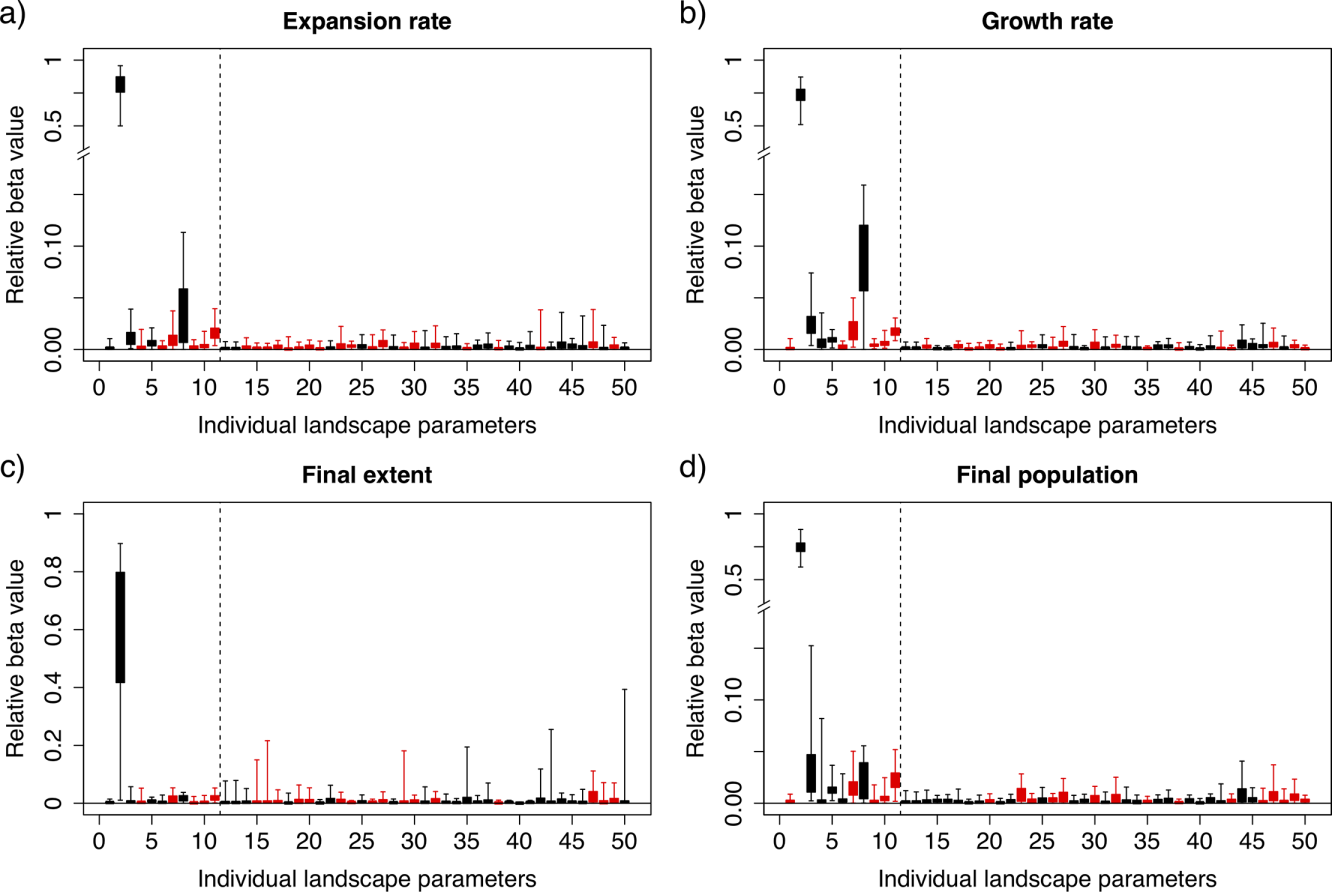
Plots of the relative importance values (absolute value of the *β* coefficients, but those shown in red have negative average coefficients) of each landscape characteristic for predicting each of the invasion response metrics across all invasiveness trait combinations. Boxes indicate the interquartile ranges and whiskers the full ranges of *β* values across all invader trait combinations. The dashed vertical line separates parameters that describe landscape composition (1–11) or spatial structure (12–50). Note that in panels a, b, and d the break and change in scale on the y-axis. In each of these cases the *β* values for the abundance of grassland were much higher than any other parameter and so we have separated it to increase the visibility of other parameters. The order of landscape characteristics is the same as in Table 1.

Once the optimal β coefficients were selected for each combination of invasion metric and invader traits, we were able to compare the strength of the relationship between different landscape metrics and the relevant aspect of invasiveness. Because all covariates were centered and scaled, the relative size of different β coefficients offers a direct comparison of importance of the specific landscape characteristics on the response. Then, by scaling β values relative to the strongest predictor, comparisons could also be made across invader trait scenarios. Overall, we found that abundance of high-quality habitat (grasslands) in the given landscape was the strongest predictor of invasion, providing on average 70–80% of the predictive power of the model for any of the invasion metrics. In contrast, most other landscape characteristics had nuanced and contingent effects; we observed significant variability in prediction value of landscape characteristics between response metrics (both between short and long time scales and population growth versus spatial extent of invasion) and under different invader trait scenarios, seen in the spread of values in Fig 3A–3D.

**Table 1:** List landscape parameters analyzed and order in Figs 3&4.

Position	Landscape parameter	Landscape aspect
1	Proportion unsuitable	Composition
2	Proportion grassland	Composition
3	Proportion deciduous forest	Composition
4	Proportion coniferous forest	Composition
5	Proportion mixed forest	Composition
6	Proportion pastureland	Composition
7	Proportion cultivated cropland	Composition
8	Proportion road	Composition
9	Proportion wetland	Composition
10	Proportion open water	Composition
11	Proportion shrubland	Composition
12	Number of Patches	Landscape heterogeneity
13	Patch density	Landscape heterogeneity
14	Largest patch index	Landscape heterogeneity
15	Total edges	Landscape heterogeneity
16	Edge density	Landscape heterogeneity
17	Patch area: area-weighted mean	Landscape heterogeneity
18	Patch area: median	Landscape heterogeneity
19	Patch area: standard deviation	Landscape heterogeneity
20	Radius of gyration: area-weighted mean	Landscape heterogeneity and patch shape
21	Radius of gyration: median	Landscape heterogeneity and patch shape
22	Radius of gyration: standard deviation	Landscape heterogeneity and patch shape
23	Shape index: area-weighted mean	Patch shape
24	Shape index: median	Patch shape
25	Shape index: standard deviation	Patch shape
26	Fractal index: area-weighted mean	Patch shape
27	Fractal index: median	Patch shape
28	Fractal index: standard deviation	Patch shape
29	Perimeter to area ratio: area-weighted mean	Patch shape
30	Perimeter to area ratio: median	Patch shape
31	Perimeter to area ratio: standard deviation	Patch shape
32	Circumscribing circle: area-weighted mean	Patch shape
33	Circumscribing circle: median	Patch shape
34	Circumscribing circle: standard deviation	Patch shape
35	Contiguity index: area-weighted mean	Patch shape
36	Contiguity index: median	Patch shape
37	Contiguity index: standard deviation	Patch shape
38	Perimeter area fractal dimension	Patch shape
39	Euclidean nearest neighbor: area-weighted mean	Patch connectivity or isolation
40	Euclidean nearest neighbor: median	Patch connectivity or isolation
41	Euclidean nearest neighbor: standard deviation	Patch connectivity or isolation
42	Contagion	Patch aggregation
43	Percentage of like adjacencies	Patch aggregation
44	Interspersion and juxtaposition index	Patch aggregation
45	Patch Richness	Habitat diversity
46	Shannon's diversity index	Habitat diversity
47	Simpson's diversity index	Habitat diversity
48	Shannon's evenness index	Habitat diversity
49	Simpson's evenness index	Habitat diversity
50	Aggregation index	Patch connectivity or isolation

On average across all invader trait scenarios, few landscape characteristics have more than a marginal impact on invasion dynamics. However, for certain scenarios and response metrics, many landscape characteristics have larger effects (Fig 3) and provide up to 33% of the predictive power of the model (e.g., in the case of the area-weighted mean of patch contiguity for predicting transient expansion rates). After grassland abundance, the most informative landscape composition characteristics were abundance of deciduous forest, coniferous forest, and roads (all of which increased invasions), and the abundance of cropland, which reduced invasions. The most informative spatial characteristics were area-weighted mean of patch perimeter to area ratio, area-weighted mean of patch contiguity, total edge number, and edge density, all of which reduced invasions. Additionally, the final population metric was strongly influenced in a number of scenarios by the number of patches, patch density, percentage of like-adjacencies and aggregation index which all increased population sizes.

**Fig 4:**
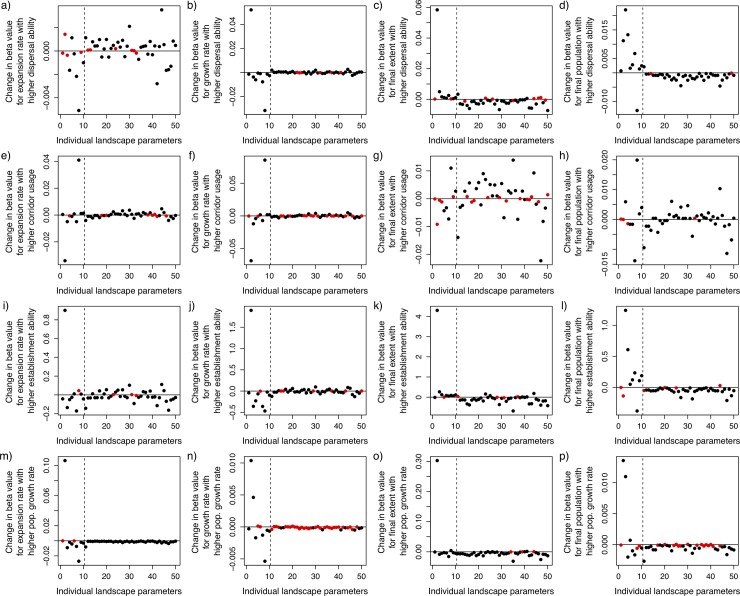
Estimates of the change in importance of each landscape characteristic as invasiveness levels change. Values plotted are the mean coefficients (*Φ*) predicted by the Bayesian linear model for each invasion response metric (expansion rate in the first panel of each row, growth rate in the second, final extent in the third and final population in the fourth as in Fig 1) and with respect to changes in each invasiveness trait (dispersal ability in the top row, corridor usage in the second, establishment ability in the third, and population growth rate in the fourth). Landscape characteristics that showed significant changes (95% confidence interval of the estimate did not include 0) are plotted in red and non-significant values are plotted in black. The order of landscape characteristics is the same as in Table 1 and Fig 3.

We observed dependent effects of certain landscape characteristics, contingent upon certain plant invasiveness traits. Such dependencies result from interactions between these factors. Observed interactions are detailed below for each individual invasiveness trait, but overall the observed patterns for dispersal strength, establishment ability, and population growth rate were generally similar, and contrasted to observed patterns for the corridor usage trait (Fig 4, and see S1–S4 Tables for specific values for each parameter). For the former traits, the direction of change (as invasiveness traits increased) in predictive value of landscape metrics typically was different for composition and spatial structure characteristics (i.e., when the importance of spatial metrics increased, the importance of compositional metrics decreased); however, there are notable exceptions, in the cases of abundance of grasslands and, to a lesser degree, the abundance of roads. Additionally, interaction patterns tended to be similar between analyses using the two metrics of transient dynamics (30 year average expansion rate and 30 year average population growth rate) and the two metrics of long-term outcomes (final invasion extent and final population size). For dispersal strength, establishment ability and population growth rate, the importance of landscape compositional characteristics tended to decrease with stronger invasiveness for transient dynamics, while their importance increased for long-term metrics. In contrast, the changes in importance of spatial structure characteristics were more diverse, but broadly tended to increase in importance with stronger invasive traits for transient measures of invasion, but decrease in importance for long-term measures.

### Specific interactions with landscape characteristics for each plant invasiveness trait

#### Dispersal extent

On transient time scales, there were conflicting impacts of landscape spatial structure characteristics between the growth and expansion responses (Fig 4A and 4B). For both responses, the importance of spatial structure characteristics increased with stronger dispersal ability. Given high dispersal ability, invasion extent was generally higher in landscapes with characteristics that indicate higher fragmentation (many patches, high edge values, high perimeter-to-area ratio) and lower when in landscapes with fewer, larger, more regularly shaped patches (high patch area, high contagion, high contiguity, high like adjacencies). Conversely, higher invasive population growth rates were seen in the less fragmented landscapes, suggesting a trade off between characteristics that support spread through the landscapes and those that quickly allow expansion of populations once they establish (e.g., large easily accessible swaths of high quality habitat). At longer timescales (Fig 4C and 4D), there was a relatively consistent pattern for both population size and invasion extent, with compositional characteristics becoming more important as dispersal strength increases and spatial characteristics less important. As would be expected, high abundance of favorable habitat types (grassland, hay, deciduous forest) tended to produce stronger invasions while unfavorable habitat types (crop, wetland, water) limited invasions and these patterns were exacerbated as dispersal ability increased.

#### Establishment ability

Similar to dispersal extent, there was a general trend of decreasing importance of compositional characteristics with increasing establishment ability on short time scales (Fig 4E and 4F), and increasing importance in the long-term (Fig 4G and 4H). Transient dynamics showed a similar pattern across growth and expansion rates, with significantly increasing importance of characteristics relating to landscape spatial complexity (e.g., lesser effect of large, regularly shaped patches of few habitat types) when establishment ability was high. This contingency was evident in the increasing importance of the shape, circumscribing circle, patch area, and patch richness parameters, as well as numerous parameters related to the standard deviation of landscape metrics, which indicate high variability over the landscape. Over longer timescales, the characteristics that increased in importance at high levels of establishment ability were almost exclusively compositional, while spatial structure characteristics generally lost importance. Notably, however, the importance of grassland and road abundances decreased with increasing establishment ability when looking at final invasion extent but not final population size.

#### Growth

In contrast to the two traits discussed previously, relatively few landscape characteristics interacted with growth rate in their impact on invasion extent at either transient (Fig 4I) or long timescales (Fig 4K). We infer that the main effect of growth rate is a linear increase in expansion rate, and that this effect is not modulated by landscape features so their relative importance is consistent. For the few landscape characteristics that did interact with the growth rate invasiveness trait for expansion rate or invasion extent, these characteristics generally decreased in their predictive power as growth rate increased. In contrast, for predicting population growth or final population size (Fig 4J and 4L), many landscape characteristics had significant changes in importance across different levels of the growth rate trait. However, only grassland abundance had a large increase in importance, while most other characteristics decreased.

#### Corridor strength

The interaction between corridor usage and landscape characteristics showed relatively different patterns from other invasiveness traits (Fig 4M–4P). In essence, we observed a broader range of interactions with landscape characteristics, but relatively more consistent patterns across response metrics. Corridor strength showed a strong interaction with road abundance, which was expected because when invaders can use roads as corridors that significantly increased invasions. Other spatial structure characteristics that showed strong positive interactions with corridor usage generally related to patch shape. In particular, these were characteristics that relate to the presence of long, regularly shaped patches (e.g., high contiguity levels, high ratio of circumscribing circle to patch size, low interspersion), potentially also identifying the presence of roads and dispersal corridors. Additionally, the abundance of habitat types that were unusable by *Miscanthus* and likely acted as *de facto* dispersal barriers (e.g., water, wetlands) tended to increase in importance with increasing corridor strength because in those scenarios their presence or absence strongly changed the invasion dynamics. In contrast, habitat types that were relatively good for *Miscanthus* (e.g., grasslands, deciduous forest) were important to supporting invasive spread when the invaders could not use dispersal corridors, but lost predictive power when invaders could spread via corridors instead of needing regular patches of high quality habitat.

## Discussion

The magnitudes of invasions are influenced by both the particular traits of invaders and the landscapes in which those invasions occur. While certain traits can increase the invasiveness of a species, even the most effective invaders showed significant variability in spread in different landscapes. Thus, for many of the practical questions of invasive species management, the composition and spatial patterns of landscapes can play an important role [18]. Additionally, we observed very different patterns when considering different aspects of invasions (short vs. long time scales, spatial extent vs. population size), making the particular context of management or forecasting goals a critical consideration. While both plant invasiveness traits and landscape characteristics had strong and relatively consistent influences on invasion dynamics, we identified numerous interactions between the two types of drivers. These interactions showed a variety of patterns, indicating that there was not a uniform response to increasing “invasiveness” of the invader, but rather that different landscape attributes influence specific aspects of the invasion process.

### The role of simulation models in evaluating invasion risks

Our simulation modeling approach allowed us to address the role of landscapes on invasion dynamics. We were able to simulate invasions on real, remotely sensed, landscapes where the invader’s population dynamics varied among habitats; this provided the basis for evaluating complex spatial dynamics. As a stochastic variation of an integro-difference equation (IDE) model in a complex landscape, rather than an analytical model (i.e., classic IDE models), the influence of landscape structure on invasions can be seen as an emergent property of the landscape. By using a model-based approach to understanding invasion dynamics, we were able to 1) run simulations across a wide range of landscapes to produce large datasets and 2) replicate invasions in the same landscapes with invaders that have slightly different traits. This combination allowed us the opportunity to look at both how different landscape characteristics influence invasions and how the importance of particular landscape characteristics in predicting invasions changes for invaders with different ecological traits. We were also able to do this in ways that would not have been possible with experimental or observational methods. Additionally, by using a Bayesian model regularization approach [32], we were able to overcome many of the challenges of managing datasets of numerous landscape metrics, particularly those relating to highly correlated data.

Our use of dynamic simulations was also critical to identifying the influence of landscape structure on invasions as this was more clearly seen in invasion dynamics rather than long-term patterns. Much of the recent effort in predictive modeling of invasive species has been focused on identifying and then quantifying the abundance of high-quality habitat, particularly with species distribution models (SDM) or habitat suitability models (HSM) [36,37]. To a degree, our analysis supports this focus as we also found habitat quality to be the most informative predictor of invasion dynamics. However, the fact that spatial structure characteristics were also informative and that relative importance changed with ecological traits of invaders suggests that a sole focus on habitat is a limited approach for predicting invasion dynamics. Our ability to identify these more complex influences of landscapes on invasion dynamics is in part because we explicitly considered both short-term and long-term dynamics. There has been a growing appreciation that invasive species violate the equilibrium assumption embedded in SDM and HSM techniques, and approaches to adapt to this issue are being developed [37,38]. Yet, even these efforts only focus on the lack of equilibrium when determining habitat suitability; predictions made by most models still assume complete penetration of invaders and thus spatial or temporal dynamics inherently become inconsequential. For most management and decision making purposes, this is not a reasonable assumption. There is a need to consider short-term dynamics because they can have important implications for risk analysis and design of management or best practices strategies [39,40]. Short-term dynamics may be the most important determinants of whether spread can be prevented and as such may be paramount concerns in the case of potentially invasive biomass crops, as society may be unwilling to tolerate any substantial risk of widespread establishment of such species.

### Invasions are complex emergent processes with multiple drivers

The variability between multiple response metrics shows that invasions are complex processes, which cannot be described or compared by a single measure. Invasion dynamics are the emergent outcome of numerous interacting processes. This complexity makes them difficult to predict, but suggests that there may be a variety of options for management and intervention, and that a focus should be placed on different targets depending on the particular goal [41–43]. While the long-term outcome of most successful invasions is extensive spread across the entire landscape, patterns over short-term timescales might be more practically relevant to questions of management. Over short timescales, invasions will certainly be less spatially extensive, but are also more constrained within certain habitat types; identifying those differences can inform where monitoring and eradication efforts should be focused. Similarly, concerted efforts on certain landscape features (e.g., roads that act as dispersal corridors or habitat islands that can act as reservoirs) can have an outsized influence on the speed and extent of spread [44]—this insight has also influenced management strategies for marine and aquatic invasive species [45,46]. Depending on the particular ecology and/or impacts of an invader, the importance of managing for spatial spread versus population size may be very different. For example, managing dispersal of species that have little impact at low abundance may be less important than focusing control efforts on the habitat patches where populations can grow to harmful levels. In contrast, the most effective strategy for species that have strong local impacts but have low natural dispersal capability could be to prevent access to dispersal corridors. Relatedly, our results indicate that changing the strength of invasiveness traits does not influence all invasion metrics equally. As might be expected, increased dispersal ability tends to influence spatial extent of invasions more than total population size (though both increase together, Fig 1). Increasing population growth rates display the opposite pattern with stronger effects on population sizes.

In general, we observed segregation in response patterns between landscape metrics that described the composition of habitat types versus metrics related to the spatial structure. Broadly, the effects of landscape composition characteristics were generally stronger than those of spatial structure metrics, similar to patterns that have been identified by others [47]. However, we also observed that for transient dynamics, the importance of spatial structure increased as invasiveness increased (Fig 4), while that of composition decreased, this suggests that analyses without consideration of such an interaction could be misleading. Overall, this pattern indicates that at low invasiveness, landscape pattern has less influence than composition on the dynamics of invasions. This is not because of an overall cessation of invasion, as invaders are still spreading. Rather, when invasions are slow the species may be unable to explore much of the landscape and only the habitat directly adjacent to the founding colony matters. As invasiveness increases and spread is greater, the composition and arrangement of habitat begins to play a larger role; however the particular means by which invasiveness increases (e.g., through higher propagule pressure from rapid population growth, greater dispersal distances, or higher seed survival) have greater impacts in certain types of landscapes versus others. This perspective is bolstered by the fact that these patterns are reversed when long-term metrics are considered. At these time scales, invaders likely have explored the entire landscape so compositional characteristics again take primacy. This question of landscape structure and its relationship to invasions has been considered before, generally showing that heterogeneity increases invasibility [15] and that landscape characteristics that are associated with disturbance can also be indicators of invasion [48]. However, this work has been limited by the relatively few types of landscape characteristics considered and because it generally has not considered how that relationship might change for invaders with different natural histories (but see [19]).

The basis for an interaction between spatial structure and specific ecological traits of invaders is clearer when there is an explicit focus on dynamics rather than equilibrial outcomes. Growth rates, fecundity, and dispersal do not impact carrying capacities, but they can influence how efficiently invaders spread through a landscape [49]. This is the aspect of invasions that spatial structure can most strongly influence; however, we observed strong contingencies in the effects of spatial characteristics, many of which had strong effects only in concert with particular invasiveness traits, indicating that the influence of spatial context is related to each trait. For example, we saw qualitatively different patterns between invasiveness traits. The spatial characteristics whose importance changed in concert with dispersal ability were largely related to fragmentation, as might be expected. Changing establishment ability most strongly influenced the importance of characteristics related to the presence of large, regularly shaped patches. This suggests that when an invader is very good at establishing, the most effective approach for spreading is from high propagule pressure produced by large local populations. In contrast, the ability to take advantage of dispersal corridors reduces the need for high propagule pressure to support spread, provided corridors are available. Thus, the importance of high-quality habitat can be relatively lower while patch characteristics indicative of corridors (abundance of road habitat, as well as parameters indicating long, linear patch shapes) have a larger effect.

### Implications for management of invasive species

Inferences of management implications from an exploratory modeling study must be cautious. However, we highlight several implications that emerge from our results, though much additional analysis would be needed before use in practice, and these are best regarded as indications of how our approach to modeling landscape effects on invasion could provide substantial insights for management. Overall it seems apparent that the presence of high suitability habitat is the central risk factor for the spread of *Miscanthus* invasions, but spatial structure can limit—or exacerbate—the early stages of invasion, particularly in highly-invasible habitats. If preventing invasion in such habitats is important, then manipulations of landscape spatial structure can provide some measure of control. By evaluating invasion risks in individual landscapes we see that grasslands with facilitative spatial structures present the worst-case scenarios for *Miscanthus* invasion, particularly in terms of the rate of spread. We observed that many of these landscapes have very regular and consistent habitats, which may indicate that *Miscanthus* can move more efficiently through large single patches of habitat. Also, those landscapes often contain roads or other linear elements that can act as dispersal corridors. Conversely, many landscapes where spatial structure had relatively strong negative effects on rates of spread had complex spatial patterning featuring many small patches of habitat, suggesting that complex fragmented landscapes have slower spread rates. This finding suggests that management practices that create such landscapes might be useful interventions in particularly favorable landscapes for *Miscanthus* invasion, such as grasslands with large, rectangular habitat features. In practice, fragmentation might be introduced with long, narrow patches of a contrasting habitat type, e.g., so-called prairie strips that are being used to add habitat diversity to annual row-crop landscapes in Midwest US [50]. Of course, these management implications only apply specifically to Miscanthus and the invasiveness variants considered in this modeling study. Our results also argue that the influences of landscape structure vary with respect to specific traits of the invader and thus different landscape patterns may be better optimized for limiting the spread of other invasive species. Thus, it is important to recognize that manipulation of spatial structure, as a management option for limiting initial spread of invasive species, is highly tentative and requires examination across invasiveness traits and habits.

## Conclusions

In this work, we build on previous understandings that landscape characteristics can influence the spread of invasions and that invader traits impact certain aspects of the invasion process to explore interactions between those aspects of invasion dynamics. We found that the most important landscape attributes had to do with habitat type and quality, with spatial patterns playing a more subtle role. In general, the relationships we observed between particular landscape patterns and invasive traits showed large variability, but followed predictable patterns (e.g., the importance of dispersal corridors increases with the ability to use them but decreases as natural dispersal ability increases). However, we found that these interactions had strong influences on invasions in some instances, and thus should be considered in predictive or management contexts [11,51]. There has been some focus on the role of landscape spatial structure on invasions [10,11], but there has not been a concerted effort to tailor predictions to specific invaders. Our findings have emerged from assessments of landscape-trait interactions in simulations of one particular invader life-history and functional type. The pronounced interactions that we have observed warrant a broader investigation of landscape-trait interactions among a range of invader life histories and functional types.

Such inquiry could significantly advance both understanding of and predictive ability for invasions, and will further promote a shift in perspective that recognizes invasions as emergent properties of complex systems. Moreover, there is a need to increase focus on the temporal patterns of invasions rather than static predictions of suitability [52,53] and to recognize that different management concerns may need to consider different aspects of invasions. These approaches likely will be more intensive—in terms of information, effort, and computation—than current models, particularly when extended to broader spatial scales and a wider range of invader types. However, if those challenges can be overcome, the prediction and management of invasions may be usefully advanced [36]. In particular, these approaches many improve management of complex invasion challenges associated with species introductions, e.g., potentially invasive biomass crops where management must focus not on eradication, but rather on maintenance of populations within constrained spatial boundaries.

## Supporting information

S1 Table Estimates for coefficients of change in landscape parameter beta values with increasing invasiveness traits for invasion expansion rate. (XLSX)

S2 Table Estimates for coefficients of change in landscape parameter beta values with increasing invasiveness traits for invasive population growth rate. (XLSX)

S3 Table Estimates for coefficients of change in landscape parameter beta values with increasing invasiveness traits for final invasion extent. (XLSX)

S4 Table Estimates for coefficients of change in landscape parameter beta values with increasing invasiveness traits for invasive population size. (XLSX)
